# Men’s awareness of cervical cancer: a qualitative study

**DOI:** 10.1186/s12905-018-0650-9

**Published:** 2018-09-24

**Authors:** Hae Won Kim, Duck Hee Kim, Youngji Kim

**Affiliations:** 1 College of Nursing, Seoul National University, 103 Daehak-ro, Jongno-gu, Seoul, 03080 Republic of Korea; 2 Department of Nursing, Woosuk University, 443, Samnye-ro, Samnye-eup, Wanju-gun, Jeollabuk-do 55338 Republic of Korea; 3 College of Nursing, Gachon University, 191 Hambakmoero, Yeonsu-gu, Incheon, 21936 Republic of Korea

**Keywords:** Awareness, Cervical cancer, Interview, Qualitative research

## Abstract

**Backgrounds:**

As HPV is a sexually transmitted virus, men are crucial in the prevention of cervical cancer, but research about men’s awareness on cervical cancer is limited. Therefore, in this study, we investigated men’s awareness toward women’s cervical cancer, to thoroughly understand the viewpoints of men, and to emphasize the centrality of the role of men in the prevention of cervical cancer.

**Methods:**

A qualitative descriptive design was chosen to explore men’s awareness of women’s cervical cancer. Twelve men aged 20–58 were interviewed. Snowball sampling was conducted to recruit participants.

**Results:**

Most participants stated that they were not interested in women’s health, and that they did not have much knowledge about cause and prevention of cervical cancer. They acknowledged that cervical cancer was different from other cancers, based on cause and prognosis of disease. The recognition of cervical cancer in participants varied widely depending on their relationship with women. Respondents’ recognition of cervical cancer was classified into four types based on a Situational Awareness (SA) model including individual factors (knowledge about cervical cancer, interest in women’s health) and system/task factors (relationship with women, men’s responsibility).

**Conclusion:**

This study is one of the few studies describing men’s awareness on cervical cancer. Korean men’s awareness level was low, and their concern and knowledge were not good. Some participants thought that cervical cancer can be treated, can be prevented, and is recognized as a disease of a female with men intervening. Our participants perceived that the role of men is necessary for the prevention of cervical cancer. Therefore, a strategy is needed to develop the awareness and knowledge of men on cervical cancer prevention. When planning cervical cancer education for men, phase and type-specific approaches are required, depending on perception level.

## Background

Cervical cancer is the seventh most common cancer, with 528,000 newly diagnosed patients worldwide in 2012 [1]. Approximately 270,000 women died from cervical cancer in 2016 [2]. According to the cancer incidence published in 2017, 214,701 cancer cases occurred in Korea in 2015, including 3582 cases of cervical cancer. This finding excluded intra-epithelial cancer. Cervical cancer accounts for 1.7% of all cancers and is considered the seventh most frequently occurring cancer among females [3]. Korea has been conducting cancer screening since 2002 in order to more quickly detect and treat cancer, which is the leading cause of death in Korea. Currently, five major cancer screening projects, including cervical cancer screening, are underway. Although Korea supports the national screening costs for the five most common cancers, the rate of cervical cancer screening in 2013 was only 37.4%, which was insufficient for a cost-effective approach in preventing cancer [4].

The primary cause of cervical cancer is HPV infection and this is acquired mainly through sexual activity [5]. There is a serious risk for women that HPV infection may become chronic, and pre-cancerous lesions progress to invasive cervical cancer [6]. Approximately 70% of cervical cancer can be prevented by HPV vaccination, and cervical cancer can be found early by rapid cervicovaginal smear screening [7].

Korean vaccination with HPV started for females aged 12–13 on June 1, 2016 through National Immunization Program [8]. HPV vaccination for cervical cancer prevention projects has been focused mainly on women [9]. Men are excluded, both young boys age 12–13 who are (mostly) not sexually active as well as men, from the prevention of cervical cancer.

The age at which sexual intercourse is starting is lower and the influx of open sexual culture has brought many changes, so more than 43 countries have implemented HPV inoculation for national vaccination. Of these countries, gender-neutral vaccination has been recommended in the US, Canada, Austria, and Australia [10]. However, in the US, the initial policy was to vaccinate women only, for two primary reasons: the vaccine was promoted as preventing cervical cancer, a female disease, rather than HPV infection, an STD affecting both genders; and religious conservatives objected to the vaccine as they felt it promoted sexual promiscuity Coverage of males, even with the newer bivalent vaccine, differs by state and remains low (around 13%) and controversial because of costs [11].

Because the participation rate of women in cervical cancer screening is not high, variable approaches, such as pandemic chastity, and increased awareness of sex health in both men and women are needed to prevent spreading of cervical cancer. At this time, the active involvement of men in cervical cancer prevention is necessary. The WHO has suggested items that men should be aware of to help prevent cervical cancer. The roles of men are divided into direct preventive roles and indirect preventive roles such as avoiding cancer-causing substances, ceasing smoking, limiting the number of sex partners, and using condoms during coitus [12].

The role of men in the prevention of cervical cancer is crucial, and awareness toward cervical cancer is expected to affect men’s health behavior and attitude. Men have low awareness of cervical cancer and low interest in prevention of cervical cancer [13]. Research is sparse on male awareness of women’s cervical cancer and why men were excluded from prevention strategies worldwide, including the Republic of Korea [14, 15]. Most research focused on risk factors of cervical cancer, screening, and HPV vaccination in women [16, 17]. Because cognitive changes must be preceded by positive behavioural changes, we need to be aware of the perception of men on cervical cancer. Qualitative research is based on unique methodological traditions that describe social or human issues. A qualitative study will provide a comprehensive summary of cervical cancer awareness among men and help health professionals better understand this phenomenon by understanding male perceptions. To identify factors that affect cervical cancer prevention behaviours of men, we should conduct qualitative investigations to discover and understand new factors.

Therefore, in this study, we investigated men’s awareness toward women’s cervical cancer, to thoroughly understand the viewpoints of men, and to emphasize the centrality of the role of men in the prevention of cervical cancer.

## Methods

This is a descriptive qualitative study to investigate the men’s perception and specific views about women’s cervical cancer. We considered age dispersion of men in the sample selection, under the assumption that generational differences might reflect Korean men’s awareness of our participants. Thus other demographics such as education or job were not considered in this qualitative study. Participant selection was designed to include participants of all ages.

### Theoretical framework

During the interviews, efforts were made to obtain and saturate data on cervical cancer-related perceptions of men, taking into account the various demographic and social backgrounds (age, marriage) and family relationships. However, in the interview process, we found that the perceptions and attitudes of men varied according to backgrounds and characteristics, and perceptions consisted of a complex spectrum. Therefore, we borrowed the Situational Awareness (SA) Model to organize the perceptions of men within a more complex and sophisticated structure [18].

The SA Model is the theoretical framework for the discussion in this study. SA was applied to aviation systems, and is used in decision making, safety, behavioral science, nursing and public health care [19]. SA refers to awareness of surroundings, and is presented as three dimensions: perception, comprehension, and prediction [14]. A person’s perception of the elements in their environment forms the basis of SA. SA will affect person’s decisions and actions. Two major factors influence decision making process and performance. First, individual factors, knowledge and attention, vary in human SA. Second, task/system factors including workload, stress, and complexity may alter SA [18].

### Participants

Participants of this study were 12 adult Korean men aged between 20 and 58. Seven participants (58.3%) were married, and five participants (41.7%) were single. The subjects were men who lived in the city of C and the surrounding area, where the interviewer resided. The first person was indirectly referred through an acquaintance of an interviewer’s community fellowship, a church meeting, and a husband’s network and was not directly related to the interviewer. The selection criterion included men aged over 20 years. After the interview with the participants, they were selected to be introduced to other participants, and snowball sampling was conducted to recruit participants.

### Data collection

Data collection was conducted from May to August 2016. Interviews were conducted by researchers (H W K, D H K) with one observer. The place of the interview was decided upon by the participant, and we used a quiet cafe near their residence, internal church facilities, participant’s house, and lounge of a workplace, so that they could participate in the interviews comfortably.

At the beginning of the interview, we started with a general conversation that was related to everyday life and continued with open-ended questions to construct a relaxed atmosphere of trust with the participants. We said, ‘Please, tell me everything you know about cervical cancer’. We started out with a wide range of questions and then conducted interviews with detailed questions. These questions were determined through a pilot interview with two men and a discussion among researchers (Table 1).

**Table 1:** Questions and sub-questions

Questions	Sub-questions
1. Please tell me everything you know about cervical cancer.	- What do you think is the cause? - What do you know about symptoms?
2. What do you think of cervical cancer from the standpoint of men? How can you talk about cervical cancer?	
3. What do you think is the role that men can do for the prevention of cervical cancer?	- What role can you do in the home? - What role can you do in the social dimension?

The observer was a RN and had 3 years of work experience in a gynecology department. She had conducted several qualitative studies and completed Ph.D. coursework. Each participant underwent one to three interviews of 50 to 90 min. Interviews continued until new material was not obtained. The interviews were audio recorded after participants’ approval and transcribed verbatim immediately. In addition, the participant’s facial expressions and nonverbal behaviours were transcribed in the field notes. The interviews with the researchers were discussed, and the missing contents were supplemented by the next interview. We collected the data until no more new contents appeared. After interview, personal identification information was removed, and a serial number was assigned. Interviewers and other researchers cross-checked the main contents to improve their accuracy.

### Data analysis

Content analysis was designed to investigate and categorize the recorded content systematically [15], appropriate for in-depth analysis of adult men’s perception of cervical cancer. While all researchers repeatedly read transcripts and listen to the recording, they were extracting meaningful sentences, collecting similar content and categorizing them several times. In this process, they shared thoughts about the category, compared the sentences in the same category, deleted the semantically confusing categories, and created new categories. In this study, the researchers deliberated until more than six opinion and agreement exchanges took place. The meaning of each theme and subtopic was clearly generated by a continuous analysis, and themes and subtopics were named by researchers. Finally, three themes and nine subtopics were derived from our data.

The data of this study are based on the criterion of Guba and Lincoln [20]. For the rigor of this study, we examined the interview contents and research results through member checking of participants, reviewing and discussing with researchers. The understanding of the research and the assumptions and prejudices of the researchers were recorded in the reflection diary, and it was used as reflective data during the data collection and analysis period.

### Ethical consideration

The study received ethical approval from Institutional Review Board of Seoul National University. Researchers directly explained the purpose and method of the research to the participants, confirmed that they voluntarily agreed to participate, and conducted interviews. We explained the option of refusal to participate in the study at any time during the interview and assured the participants of anonymity. After the interview, participants were given compensation for participating in the study (approximately 45,000 Korean Won gift certificate).

## Results

The demographic characteristics of participants are shown in Table 1. Half of the participants were married (aged between 20 and 58 years, mean = 37.3, SD =11.6), and most were university graduates (*n* = 11, 91.7%). Their religions were mostly Protestant (41.7%) and Catholic (33.3%) (Table 2).

**Table 2:** Characteristics of participants (*N* = 12)

Characteristics	Frequency	Percent (%)
Age (years)	37.3 ±4.5	
20–29	3	25.0
30–39	4	33.3
40–49	3	25.0
50–59	2	16.7
Marital Status		
Married/partner	7	58.3
Single	5	41.7
Economic Status		
High	1	8.3
Middle	8	66.7
Low	3	25.0
Educational attainment		
High school	1	8.3
University	11	91.7
Religion		
Protestant	5	41.7
Catholic	4	33.3
None	3	25.0

There were two themes (lack of knowledge and interest about cervical cancer, a common-sense level of understanding) in awareness of cervical cancer, two themes (lack of knowledge and interest about cervical cancer prevention, insufficiency of understanding) of cervical cancer prevention, and five themes on differentiation of cervical cancer from other cancers (Table 3).

**Table 3:** Korean men’s recognition of cervical cancer and prevention

Themes	subtopics
Awareness toward cervical cancer	Inadequate knowledge and interest
	a common-sense level of understanding
Awareness toward cervical cancer prevention	Inadequate knowledge and interest
	Insufficiency of understanding
Awareness toward difference from other cancer	Recognized as curable disease
	Special disease related to the reproductive organ
	Recognized as sexually transmitted disease
	Recognized as preventable disease
	Absence of prejudice against cervical cancer

### Theme 1: Awareness on cervical cancer

#### Inadequate knowledge and interest to cervical cancer

Participants stated that they were not interested in women’s health or did not know about it, and they also said that they did not have much knowledge about the cause of cervical cancer.

*I didn’t have any reason to be interested in cervical cancer, so I don’t know about it (cause of cervical cancer) that much. Not that special, but I think it seems similar to breast cancer. I haven’t thought about it much (Respondent 1)*

*I don’t know about [cervical cancer]. I do not know detailed information about the causes of cervical cancer (about human papilloma virus (HPV). No, I haven’t heard about it before (Respondent 5)*

#### A common-sense level of understanding

Some respondents who had a common-sense level of understanding about the cause of cervical cancer answered as follows:

*At first, I knew that cervical cancer is one of the diseases of women, but one day by chance I read an Internet article about it, and I knew that the main route that women got cervical cancer was through men. Men tend to be more promiscuous, and when a man transmits a sexually transmitted disease to a woman, that becomes cervical cancer. So men rather than women need a vaccination. That’s all I know (Respondent 6)*

*(About human papilloma virus (HPV), the cause of cervical cancer) I was aware of it before. I saw some health campaign materials about it. Cervical cancer is not an issue for women only, but instead, it can occur in some kind of a relationship (embarrassed) with men. I don’t know how to talk about it. Anyway, I heard that men also need a vaccination (Respondent 4)*

The only respondent who explained the cause of cervical cancer accurately was a man who majored in biological engineering in college who was in a relationship, and he said that he was vaccinated against HPV.

*As far as I know, the exact cause is still unknown, but HPV is found in the uterine cervix of cervical cancer patients. I heard over 95% of people are exposed to the virus (Respondent 3)*

### Theme 2: Awareness of cervical cancer prevention

#### Inadequate knowledge and interest to cervical cancer prevention

To the question about how to prevent cervical cancer, men responded based on their understanding. Those who initially lacked interest did not know much about cervical cancer, and said that they did not know about prevention, answering the question uncertainly based on information they heard from others.

*Um, of course, they need to get a vaccination. (Except that) I don’t know…… I think I saw some people got a shot in the health center in my college, but I don’t know about it in detail. I am not very aware of cervical cancer, so I don’t know what kinds of prevention there are (Respondent 1)*

The one respondent who had accurate knowledge about cervical cancer and had a vaccination said that there were two methods for prevention: first, both a male and female get a vaccination before having sexual relations, and second, both use contraceptive devices (etc. condom).

*One simple way is to use contraceptive devices, and both a man and woman should get a vaccination before having sexual contact. After getting a shot, well, I haven’t thought about it further, and I don’t know about it more (Respondent 3)*

#### Insufficiency of understanding

Those who had information on women’s health and cervical cancer at least partially mentioned sexual history from a man’s perspective, but as the interview went on, they could not explain it in detail or set forth their understanding since they felt embarrassed to talk about the topic. They tried to end the conversation with a simple answer or answered the question carefully but highlighted that they did not know well.

*First, men should be sexually clean. That’s it (Respondent 6)*

*The most important thing is, the surest way will be not having it (a sexual relation). But isn’t it impossible? It is impossible not to have sexual relations at all. So the most viable way will be absolutely vaccination (Respondent 3)*

### Theme 3: Difference in awareness of cervical cancer and other cancers

Participants are not very knowledgeable about cervical cancer, but they think it is preventable and curable, unlike other cancer. One participant thought that cervical cancer is more serious than any other cancer. Some participants believed that cervical cancer was related to the uterus and that it can be transmitted by sexual intercourse.

#### Survival rate and cure rate: Recognized as curable disease

Most participants stated that cervical cancer’ survival rate and cure rate is high. One participant thought cervical cancer was more serious *(Respondent 2).*

*…… Compared to other cancers, I think its survival rate is high, and its treatment rate and cure rate are high. (Respondent 4)*

*One thing I remember is that the prognosis is not that good, and it requires aggressive treatment. As I remember, compared to other cancers, its lymph nodes are excised more aggressive (Respondent 2.)*

#### Related body parts: Special disease related to the reproductive organ

Cervical cancer has been recognized as a special disease because it is related to the uterus, women’s reproductive organ.

*I don’t know in detail, but, um, general diseases such as colorectal cancer and stomach cancer are all related to digestive organs, cancers like uterine are related to reproductive organs. That’s my opinion (Respondent 5).*

#### Cause of occurrence: Recognized as sexually transmitted disease

Participants recognized cervical cancer as a unique disease because it is caused by a virus and is a sexually transmitted disease.

*Others are caused by genetic causes, and cervical cancer is affected by environmental conditions (Respondent 6).*

*First of all, the key cause is sexual transmission, so it is not enough that only women are careful. I think men should do something, so I think men should have knowledge about this. I don’t know about causes of other cancers, but I think cervical cancer is somehow a unique case (Respondent 7)*

*Of course, there is a difference. There are absolutely differences in causes or symptoms. In the cases like cervical cancer, and especially liver cancer are caused by viruses, and there’s a chance that they can occur through sexual activities. The thing that cervical cancer is distinguished from other cancers is, simply thinking, that both men and women should be careful, and this should be seen as an individual matter. I mean this is not a thing to be done alone. And men tend to be more promiscuous than women. A lot more (Respondent 3)*

*First of all, men should pay special care about diseases that can be transmitted to women, especially sexually transmitted diseases, and keep clean (Respondent 5).*

*First, men should not be promiscuous, and care about personal hygiene. And that’s all. ~~ (trying to end the conversation) (Respondent 6).*

#### Preventability: Recognized as preventable disease

Some participants stated that cervical cancer can be prevented since the development of vaccines. This was recognized as a major difference from other cancers.

*The difference would be that this can be surely preventable? Being aware of the existence of this disease, and if person use condom or protective method (vaccination), then isn’t it certainly preventable? (Respondent 1).*

#### Absence of prejudices against cervical cancer

Most Korean men are not prejudiced against women with cervical cancer due to possibility of multiple sexual partners and early intercourse. They acknowledged that cervical cancer is associated with sexual life, and even though it is related to sexual transmission, it is recognized as well as cancer of other organs.

*Not particularly about cervical cancer, it is a kind of cancer, so I guess it might be similar to other cancers (Respondent 9).*

*I mean I don’t have negative thoughts. Rather, like breast cancer and for men, lung cancer, stomach cancer, I think any woman can get that cancer, but not that easily (Respondent 4).*

*When a woman has it, she might have had sexual relations with multiple men, or if she has only one sexual partner, then he could have had sexual relations with multiple women, as I learned. But even if I hear that someone has cervical cancer, I might not think about it that far (Respondent 2)*

### Types of awareness level regarding cervical cancer

Based on our results, we identified participants’ types of awareness of women’s cervical cancer by men’s engagement into women’s issue, their knowledge, their interest in women’s health, and relations with women (Table 4). The recognition of cervical cancer in participants in this study was highly affected by their relations with women.

**Table 4:** Types of men’s recognition of women’s health and cervical cancer

Type of recognition	Respondent	Engagement in women-related issues	Interest in women’s health	Knowledge about cervical cancer	Relation with women	Men’s responsibility
Rational engagement	3,6,12	high	+	+	+	+
Irrational ignorance	5,9,10,11	middle	+	±	–	±
Irrational indifference	2,4	low	+	+	–	–
Complete indifference	1,7,8	Very low	–	–	–	–

Respondents’ recognition of women’s cervical cancer was classified into four types based on the SA model. Individual factors include knowledge about cervical cancer and interest in women’s health, and system/task factors were relation with women and men’s responsibility.

#### Type1: Rational engagement

Respondents of this type said that their interest started from their concerns about the health of family members or girlfriends, and their statements were mainly focused on related health issues. They said that they obtained relevant knowledge on cervical cancer from their academic background or online media campaigns.

*I remember that I heard that when men acquire the virus through sexual relations with others, the virus can be transmitted to other women. I just heard that it spreads. This happens because of me (men), so that we need to be careful about it. Because in the end, this will leave a scar not just on the mind, but also on the body (Respondent 12).*

#### Type 2: Irrational engagement

Some said that their close family members or brothers were very interested in women’s health, and actively intervened in and paid attention to general health problems. To the question about the cause and prevention methods of cervical cancer, however, they said that they had not thought about the area further, or that they had no idea about it.

*Men’s responsibility is big. Women are also responsible for it, but if women are victims, then women should be careful first, and women should follow prevention methods. Men do not help, so that women should care for themselves first, and once a social consensus for the importance of prevention is formed, men will feel the same way (Respondent 10).*

#### Type 3: Irrational indifference

Some respondents obtained knowledge about cervical cancer from special courses in college, and some had abundant knowledge about cervical cancer through a variety of media and proactive attention to women’s health as they got married and became concerned about their wife’s health.

*I have known about cervical cancer and received information on it from media campaigns for cervical cancer prevention. I don’t know in detail, but I heard people should get the shot at least three times before a certain age. That’s all (Respondent 4).*

*When a woman has it, she might have had sexual relations with multiple men, or if she has only one sexual partner, then he could have had sexual relations with multiple women, as I learned. But even if someone has cervical cancer, I might not think about it that far (the cause of cervical cancer is her or his indiscreet sexual relations) (Respondent 2).*

However, most of these respondents answered that their abundant knowledge of a professional level existed just as knowledge, but that they had not actively tried to think about their own roles and responsibilities, considering that the main cause of cervical cancer could be men’s indiscreet sexual relations. They answered that this was a women’s issue so that they had not thought about their roles and responsibility.

*I haven’t given much thought to it. Anyway that is women’s disease. No, I haven’t given much thought to it (Respondent 2).*

*I mean I don’t have negative thoughts. Rather, like breast cancer and for men, lung cancer, stomach cancer, I think any woman can get that cancer, but not that easily. (Respondent 4)*

#### Type 4: Complete indifference

Among the respondents who were not married, those who had no sibling or girlfriend were found to have almost no interest in women’s issues, and they said that they had not thought about women’s health especially. The absence of interest in women’s issues resulted in no attention to knowledge about cervical cancer or prevention methods. They also carelessly answered the questions that the researcher asked. As a result, ignorance about the cause of cervical cancer did not affect the roles of men in women’s health or their sense of responsibility for the occurrence of cervical cancer at all.

*Uh~ (embarrassed) I haven’t paid attention to health~~~I overheard before that women need to go to see an obstetrician regularly, but I just thought they might need to go to see a doctor occasionally. That’s all. I haven’t paid much attention to what kinds of examinations they should get and how and how regularly they should do so (Respondent 7).*

## Discussion

This study was designed to present a comprehensive summary the perception of Korean men towards cervical cancer. The study results indicated that Korean men did not have much interest or knowledge regarding the cervical cancer and women’s health, and pointed towards perception types of men.

The majority of respondents did not understand the causes of cervical cancer accurately, and lacked knowledge of prevention methods. Men’s perception of cervical cancer has not been described in detail in existing literature. Le et al. [16] found that men had a low level of perception towards HPV as a cause of cervical cancer and its sexual transmission. One researcher explored whether fathers would be willing to vaccinate their children against HPV, and indicated that sons remained ignorant of HPV or HPV vaccination [17]. In a study of women’s perception towards cervical cancer, the perception level of the women was low along with their level of knowledge, emphasizing the importance of communicating accurate information [21].

Our study found a lack of interest in cervical cancer by men. The respondents did not know of the causes or prevention methods of cervical cancer. The source of most information for respondents was the internet (Respondent 6) and campaigns (Respondent 4). It appears necessary to utilize the internet and mass media to access the general population of men to spread information about cervical cancer. Moreover, it appears necessary to explore whether health education regarding cervical cancer is directed only at females, and whether male education has been sufficiently considered [22]. Throughout the interviews, when asked about how cervical cancer can be prevented, the respondents tended to hesitate and be flustered when responding on topics relating to sex such as abstinence or sexual intercourse, hastily giving short answers. It appears that the tendency of men to be less active during discussions and conversations relating to sex would hinder cervical cancer education.

When asked about the difference between cervical cancer and other forms of cancer, our participants had a different perception of cervical cancer compared to others. As cervical cancer is transmitted through sexual intercourse, occurs in the women’s reproductive organ, the uterus, and could be prevented through vaccines, it was perceived as a specific cancer different from other forms of cancers. Our participants perceived the survival rate and rate of full recovery from cervical cancer to be very high, and perceived the management of cervical cancer to be very positive. The observation that there is a large role and responsibility for men to prevent cervical cancer (Respondents 1, 3, 5, 7) is a positive sign for prevention of cervical cancer. They perceived cervical cancer as a condition where both men and women are involved, and as a special disorder that is different from other cancers. They sympathized with the point that efforts must be made by both genders as it was being perceived as a disorder that is mediated by men, and not one that is strictly confined to women. As such, providing accurate and appropriate information to men is expected to lead to male participation in cervical cancer prevention.

FitzGerald et al. [23] found that young men tended to resist HPV vaccine and perceive it negatively. Our participants lacked knowledge about cervical cancer, leading to a lack of understanding of its causes, which then led to a lack of misunderstanding or prejudice against the carriers of cervical cancer, as cervical cancer is carried by sexual contact. The lack of stigma or prejudice against cervical cancer patients from Korean men, as well as their positive attitude towards cervical cancer management, are positive factors towards cervical cancer prevention and education.

Based on the situational awareness model, we identified four types of perspectives regarding women’s health and cervical cancer. Men’s situational awareness affects decision-making and performance. In our study, their situational awareness level remained at the situation-perception level and did not lead to decision-making and action. This study has found that while the perception of Korean men towards cervical cancer has been positive, the majority of them were at a stage of irrational ignorance, where respondents were positive but remained the perception of reality (not decision making level) or states of indifference which are irrational indifference or complete indifference. Few respondents were of the rational engagement type, where their perception had reached decision and action levels.

A past study has found that while the HPV-related knowledge of young male college students was high, it did not lead to preventive behavior [24]. Even if related knowledge increases from education on cervical cancer prevention, it is important for perception towards cervical cancer to change simultaneously. Active behavior for cervical cancer prevention would occur if men’s perception is developed towards a full understanding of the present status, and towards future projections.

Moreover, as individual and situational factors may intervene to act as hindering factors, this study presents the following recommendations. As a part of individual factors, male-centric strategic intervention that focuses on the levels of knowledge and interest are required. From the aspect of task/system factors, it is necessary to include the HPV vaccinations for men on the national vaccinations, leading to active participation by men.

In our study, we found that in general, men thought that they were not very helpful in cervical cancer prevention, and interestingly, methods of cervical cancer prevention, other than the vaccine, were not mentioned in the interviews with men. There are many ways to prevent cervical cancer, such as condom use, monogamy, and chastity [25]. However, men generally have a low perception of, and lack knowledge of, such measures. In addition to the prevention of cervical cancer, vaccination of men with cervical cancer vaccines has the advantage of effectively preventing the development of HPV-related cancers in men (genital cancer, nasopharyngeal cancer, head and neck cancer) and benefits the partner as it can help with cervical cancer prevention. This needs to be actively promoted and emphasized to the public, and the role of men in the prevention of cervical cancer should also be emphasized more.

As a result of our study, we recommend health education strategies providing men with cervical cancer prevention. When planning cervical cancer education for men, phase and type-specific approaches are required depending on the level of perception. Participants who lack interest require a strategy to raise their interest level; participants who do not have numerous relationships with women or who lack a sense of responsibility require a type of education that emphasizes the need for cervical cancer prevention in linkage with men. To switch the perception of men for them to actively intervene and participate in the prevention of cervical cancer, the educational objectives must focus on changing the perception instead of those that relate to simple knowledge of cervical cancer or of HPV vaccinations. To change perceptions, it is important for an in-depth knowledge transfer of the causes, transmissions and prevention of cervical cancer, as well as education programs that emphasize male participation and responsibility in cervical cancer prevention [26, 27]. Moreover, it appears that strategies to utilize male role models in mass media or campaigns would be beneficial for the effectiveness of information communication.

### Strengths and limitations

This study offers important contributions to the literature; however, there are important limitations that affect external validity, particularly generalizability: (1) the small sample size; (2) many of the participants were Christians; and (3) about 55% were highly educated (although this may have increased richness of data, it may have resulted in potential bias). Further, the convenience sampling employed in this study may have resulted in the selection of a specific subset of Korean men and may not have captured the extent of the diversity of men’s perspectives in general population. Therefore, these findings may not transfer to other settings or populations (e.g., not educated or individuals of other religion), or to people in non-Asian contexts.

Nonetheless, our findings can be used to develop and implement targeted health messaging, education programs and interventions focused on alleviating the cervical cancer burden. Additionally, this study is significant as it has conducted interviews to men in varying age groups from men in their twenties to their sixties, focusing on men of all ages. The findings reported here revealed the perspective of men. To date, the prevention of cervical cancer was mainly recognized as a problem only affecting women. Through this research, responsibility for this problem will be expanded both men and women. Furthermore, the study did not systematically examine the perspectives of men according to their characteristics. Future qualitative research exploring men’s perspectives and experiences for cervical cancer, as well as differences based on men’s backgrounds, would likely enhance care for cervical cancer prevention.

## Conclusion

This study is one of the few studies describing men’s awareness on cervical cancer. Korean men’s awareness level was low, and their concern and knowledge were not good. Some participants thought that cervical cancer can be treated, can be prevented, and is recognized as a disease of a female with men intervening. Our participants perceived that the role of men is necessary for the prevention of cervical cancer. Therefore, a strategy is needed to develop the awareness and knowledge of men on cervical cancer prevention. When planning cervical cancer education for men, phase and type-specific approaches are required, depending on perception level.

## Abbreviations

HPV: Human papilloma virus
SA: Situational Awareness
